# Prediction of Total Soluble Solids in Apricot Using Adaptive Boosting Ensemble Model Combined with NIR and High-Frequency UVE-Selected Variables

**DOI:** 10.3390/molecules30071543

**Published:** 2025-03-30

**Authors:** Feng Gao, Yage Xing, Jialong Li, Lin Guo, Yiye Sun, Wen Shi, Leiming Yuan

**Affiliations:** 1 College of Horticulture and Forestry, Tarim University, Alar, Xinjiang 843300, China; 15950516317@163.com (F.G.);; 2 Department of Physics, Hong Kong Baptist University, Kowloon Tong, Hong Kong, China; 3 Xinjiang Production & Construction Corps, Key Laboratory of Facility Agriculture, Alar, Xinjiang 843300, China; 4 Instrumental Analysis Center, Tarim University, Alar, Xinjiang 843300, China; 5 College of Electrical and Electronic Engineering, Wenzhou University, Wenzhou 325035, China

**Keywords:** spectral analysis, total soluble solids, ensemble learning, feature selection optimization, apricot

## Abstract

Total soluble solids (TSSs) serve as a crucial maturity indicator and quality determinant in apricots, influencing harvest timing and postharvest management decisions. This study develops an advanced framework integrating adaptive boosting (Adaboost) ensemble learning with high-frequency spectral variables selected by uninformative variable elimination (UVE) for the rapid non-destructive detection of fruit quality. Near-infrared (NIR) spectra (1000~2500 nm) were acquired and then preprocessed through robust principal component analysis (ROBPCA) for outlier detection combined with z-score normalization for spectral pretreatment. Subsequent data processes included three steps: (1) 100 continuous runs of UVE identified characteristic wavelengths, which were classified into three levels—high-frequency (≥90 times), medium-frequency (30–90 times), and low-frequency (≤30 times) subsets; (2) the development of the base optimal partial least squares regression (PLSR) models for each wavelength subset; and (3) the execution of adaptive weight optimization through the Adaboost ensemble algorithm. The experimental findings revealed the following: (1) The model established based on high-frequency wavelengths outperformed both full-spectrum model and full-characteristic wavelength model. (2) The optimized UVE-PLS-Adaboost model achieved the peak performance (R = 0.889, RMSEP = 1.267, MAE = 0.994). This research shows that the UVE-Adaboost fusion method enhances model prediction accuracy and generalization ability through multi-dimensional feature optimization and model weight allocation. The proposed framework enables the rapid, non-destructive detection of apricot TSSs and provides a reference for the quality evaluation of other fruits in agricultural applications.

## 1. Introduction

Apricots are rich in a variety of organic compounds, including polysaccharides, polyphenols, flavonoids, and organic acids, making them highly nutritious and often regarded as natural functional foods [1]. Xinjiang, particularly its southern regions, is China’s primary apricot production base, where the arid climate with low precipitation and intense sunlight creates optimal conditions for sugar accumulation in apricot fruits [2,3]. The ripening period of apricot fruits is concentrated in the high-temperature season of June. The fresh fruits are prone to decay due to increased respiration [4], and the shelf life is often less than 72 h. To extend the shelf life of fresh fruits, harvesting them at the optimal time of maturity is necessary to prevent quality deterioration due to over-maturity. Total soluble solids (TSSs) are an important indicator to evaluate the maturity of apricot fruits and serve as a key basis for the harvesting time and grading of the fruits [5,6]. Currently, the detection of TSSs in apricot fruits primarily involves destructive methods, which require the fruit to be juiced for determination. This method is not only time-consuming and labor-intensive but also results in the destruction of fruit. Therefore, a rapid and non-destructive method for detecting the TSSs of apricot fruits is needed.

In recent years, near-infrared spectroscopy (NIRS) has seen significant advancement due to its rapid, environmentally friendly, and non-invasive nature [7,8]. It has been successfully applied to the internal and external quality testing of fruits and agricultural products [9,10,11,12,13,14,15,16,17]. For example, Yuan [9] used NIRS combined with machine learning methods, extracted characteristic wavelength variables using a competitive adaptive reweighting algorithm (CARS) to establish an optimal prediction model for the bayberry TSS, and then ensembled the member model. Vega [11] employed both NIRS and Raman spectroscopy to predict the TSSs of watermelon slices, with a model RPD value of 3.06, indicating this approach can well predict the TSSs. Fass [12] collected near-infrared hyperspectral data from tomatoes and utilized machine learning to predict seven quality indicators of tomatoes. The study found that using five characteristic wavelengths can better detect tomato quality, which reduces the complexity of the model compared to full-spectrum data. Jiang [13] employed Vis/NIR spectroscopy combined with machine learning to integrate spectra from different regions of citrus fruits. The findings indicated that this combined spectral approach offers superior performance and can be used to detect citrus TSSs. Wlodarska [14] used UV, VIS, and NIR spectroscopy to detect the TSSs and TPC of strawberry fruit and juice. Yuan [15] used Vis/NIR semi-transmission spectroscopy to determine the TSSs of ‘Yunhe’ pear of a large size. Wang [16] explored the establishment of a universal detection model for apples from different origins and used Vis/NIR spectroscopy combined with CARS-PLS to predict different varieties of apples, with an R of 0.94. Seki [17] established a strawberry TSS prediction model that was not affected by strawberry color and achieved TSS predictions for white and red strawberries.

In apricot quality assessments, although NIR spectroscopy has been applied in prior studies to evaluate quality parameters such as firmness and TSSs, these approaches share the same limitations observed in other fruit studies [18,19]. Specifically, existing research has predominantly focused on developing individual regression models (such as PLSR and SVR), with efforts emphasizing optimizing preprocessing techniques, selecting relevant features (such as UVE, SPA, CARS, etc.), and fine-tuning model parameters to enhance the performance of models. However, models built on single learning algorithms often face limitations in generalization and robustness [9,15,20], which hinder their predictive accuracy. For instance, feature selection methods like UVE can be affected by random noise and PLS can be impacted by multicollinearity in spectral data. To mitigate these issues, some studies have proposed ensemble learning methods, such as weight allocation strategies across multiple models [21] and consensus strategies based on different subsets to improve prediction accuracy [22]. Adaptive boosting (AdaBoost) is known for its resistance to overfitting and typically does not necessitate intricate parameter adjustments, making it a promising algorithm for predictive modeling. Previous studies have demonstrated its effectiveness in quantitative analyses of tea leaf constituents [23] and active compounds in traditional Chinese herbal medicine [24]. However, its potential in fruit quality assessment, particularly for the non-destructive detection of fruit quality, remains underexplored. Notably, no prior research has combined AdaBoost with near-infrared (NIR) spectroscopy for apricot quality evaluation. Current applications of AdaBoost in fruit research have primarily focused on classification tasks such as defect detection [25] and quality grading [26], with few studies addressing quantitative parameter predictions. To address this gap, our study pioneers a sequential framework combining NIR spectroscopy, uninformative variable elimination (UVE), and AdaBoost ensemble learning to predict total soluble solids (TSSs) in apricots. Through the iterative selection of high–frequency, stable features and the dynamic assignment of weights to member models, the final calibration model was optimized for accurate prediction of TSSs in apricot fruits.

## 2. Materials and Methods

### 2.1. Sample Collection

Apricot samples were collected from multiple orchards within the same administrative village surrounding Aral City, Xinjiang Uygur Autonomous Region, China. This strategy ensured varietal consistency and minimized environmental variability. A random sampling approach yielded over 200 specimens, which were immediately stored in portable refrigerators to maintain biochemical stability. Samples were rigorously screened to exclude individuals with mechanical damage, pathological lesions, or signs of rot, resulting in 195 qualified experimental samples for subsequent spectral data acquisition and TSS (total soluble solid) analysis.

### 2.2. Spectral Acquisition

The near-infrared (NIR) spectrum of apricot was acquired using the Antaris II Fourier Transform Near-Infrared (FT-NIR) spectrometer (Thermo Fisher Scientific Inc., Waltham, MA, USA). Prior to data collection, the FT-NIR spectrometer was preheated for approximately 15 min to stabilize the light source. A continuous spectrum was obtained over the range of 1000 to 2500 nm. The measurement involved 16 scans and a gain setting of 4×, resulting in a total of 1557 wavenumber points.

### 2.3. Measurements of Fruit TSSs

Following spectral acquisitions, the total soluble solids (TSSs) of apricot were measured using a digital refractometer (Model: PAL-1, Atago Co., Ltd., Tokyo, Japan). Before each measurement, the refractometer prism was cleaned with pure water and dried with a lint-free cloth, followed by zero-point calibration using distilled water. TSSs of each apricot sample were then measured in triplicate: juice was extracted from individual fruits, and three independent readings were recorded. The final TSS value for each sample represents the arithmetic mean of these three measurements.

### 2.4. Spectral Calibration

#### 2.4.1. Pretreatment

During the spectral acquisition process, various methods, such as repeated measurements, light source preheating, and white and black calibration, are used to reduce the impacts of various factors on the spectral data. However, the spectral data obtained from actual measurements may still contain some interfering information that can affect the performance of subsequent predictive models. Therefore, preprocessing methods are typically employed to minimize the influence of such interfering information in the spectral data. In this study, z-score standardization was used to achieve this [27].

#### 2.4.2. Spectral Outliers

During the data acquisition stage, sample data may contain outliers due to factors such as human error and instrumental inaccuracies. These outliers can adversely affect the performance of subsequent predictive models, necessitating their removal. Robust principal component analysis (ROBPCA), a robust variant of principal component analysis (PCA) that is less sensitive to outliers than the traditional method, was used to identify these outliers [28]. Based on this results, outlier samples were removed from dataset before model development.

#### 2.4.3. Division of the Sample Set

To ensure the selection of more representative training samples and minimize human selection bias, the Kennard–Stone (KS) method was employed to divide the dataset into calibration and prediction sets. The KS method, based on the distances between samples, iteratively selects samples to ensure uniform coverage of the entire dataset [29,30]. The selected samples are more representative [31], avoiding clustering in specific regions and facilitating the development of models with enhanced generalization capabilities.

#### 2.4.4. UVE Variable Selection

The uninformative variables elimination (UVE) method is noted for its simplicity and fast computation speed [32]. It involves adding a random noise matrix to the model, performing cross-validation, and analyzing regression coefficient statistics for variable selection. This process eliminates uninformative variables, thereby reducing model complexity. Consequently, UVE has been widely adopted in spectral data processing [33,34]. However, variable selection is influenced by the added noise, leading to variability in results across runs. Addressing the selection of highly stable variables from UVE remains a challenging issue in spectral analysis.

#### 2.4.5. High-Selection Framework

**Figure 1:**
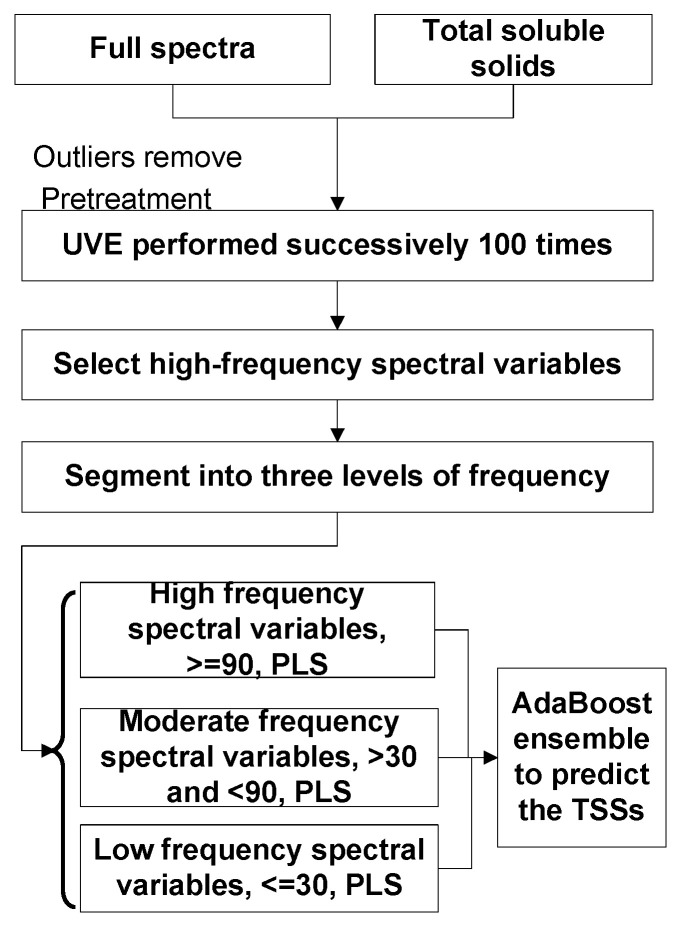
Workflow of the proposed method for NIR spectra.

During the UVE process, spectral variables with higher stability than the noise are retained. However, the noise, being randomly generated Gaussian noise, results in varying combinations of spectral variables across executions, introducing uncertainly in model predictions across different analyzers. Therefore, an ensemble framework, as depicted in Figure 1, was developed to enhance the model’s prediction accuracy and stabilize the predictions. By performing UVE 100 times, variables were selected in each run, and their selection frequency was calculated to identify spectral regions consistently selected, indicating high informativeness and stability. Variables were categorized into three levels based on their selected frequency to develop PLS models. These three PLS models served as the member model in an ensemble framework to improve the overall prediction accuracy.

#### 2.4.6. AdaBoost Ensemble

Adaptive boosting (AdaBoost) is recognized as an effective and pragmatic boosting algorithm. It begins by assigning uniform weights to all samples. Subsequently, after each training iteration, the algorithm adjusts the weights of the samples based on their performance, specifically increasing the weights of those samples that demonstrate higher error rates. This adjustment ensures that samples with elevated error rates receive greater emphasis in subsequent rounds. The iterative training process is conducted through a sequence of member models, ultimately resulting in the construction of a linear combination of the weak learners to produce the final robust learner.

#### 2.4.7. Model’s Metric

Commonly, the indicators used for evaluating regression model performance include the Root Mean Square Error of Cross-Validation (RMSECV), Root Mean Square Error of Prediction (RMSEP), Cross-Validation Correlation Coefficient (Rcv), Prediction Correlation Coefficient (Rp), Mean Absolute Error (MAE), and Systematic Prediction Bias (Bias). RMSECV quantifies cross-validation errors via root mean squared deviations, while RMSEP evaluates prediction accuracy on test sets. Rcv and Rp, respectively, measure the linear fit during validation and prediction–measurement correlations. MAE indicates average absolute prediction–observation differences, and Bias identifies systematic estimation trends for model refinement.

#### 2.4.8. Software

All procedures were simulated in MATLAB software (R2024b, Math Works Inc., Natick, MA, USA). The ROBPCA function was employed to identify the outliers with the LIBRA package [28], and the UVE method was implemented by the itoolbox package [32].

## 3. Results and Discussion

### 3.1. Spectral Analysis

**Figure 2:**
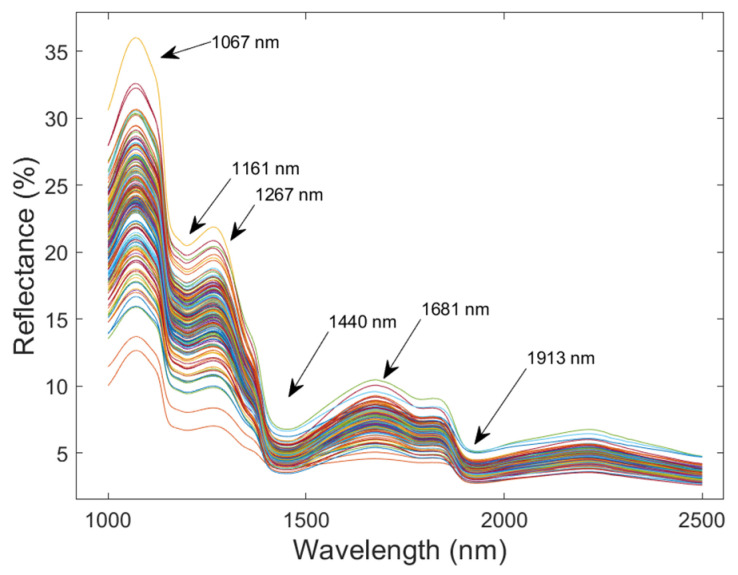
Reflectance of the raw spectra for apricot samples.

Figure 2 presents the spectral curves of apricot samples within the range of 1000 nm to 2500 nm. As can be seen from the spectral curves, although there are differences among the spectra of individual samples, the overall trend remains consistent, with no significant differences. The variations in spectral curves are closely related to the C-H, C-O, and O-H chemical bonds present in various organic substances within the apricot fruits. The 1200 nm peak corresponds to the C-H third overtone and O-H first overtone in water [8], while 1450 nm relates to O-H bending vibrations [35]. The 1060 nm peak is influenced by the broad tailing of 980 nm water absorption. The 1650 nm peak arises from the C-H first overtone, which is linked to terpenoids or other organics [36], and 1950 nm indicates C-H/C-O combination bands associated with sugars and aromatics [8]. These features collectively characterize apricot quality. Mature apricots exhibit a high TSS content averaging 17.5 °Brix [37], which may imply a greater number of C-H bonds [35]. Given that the variations in the spectral curves are related to the different chemical bonds present in the internal substances of the apricot fruits, it is feasible to employ near-infrared spectroscopy to access the internal quality of apricots [9,15].

### 3.2. Spectral Preparation

**Figure 3:**
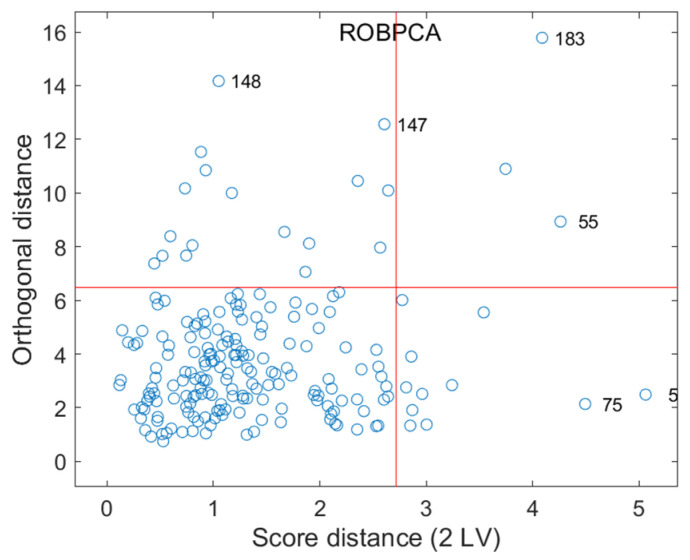
Spectral outliers detected by the ROBPCA method.

Due to the influence of measurement environment and instrumental factors, the collected spectral information may exhibit anomalies, which can affect the establishment of the model. Consequently, ROBPCA [28] was used to distinguish abnormal samples, and Figure 3 displays the map of spectral outliers, visualizing observations by projecting their orthogonal distances onto a 2D map with two principal components. Observations were categorized into four regions, with the regular predominantly located in the lower-left quadrant. At a 95% significance level, six observations were identified as spectral outliers, all of which were significantly distant from the regular cluster along the *x* or in *y* axis. Thus, a total of six abnormal samples were considered as spectral outliers, while the remaining 189 samples were considered normal and used for subsequent analysis.

Subsequently, the KS method was employed to divide the remaining samples into two subsets, resulting in 130 calibration samples and 59 prediction samples, with a ratio close to 7:3. The statistical results are shown in Table 1, where range of indicators in the calibration set basically covers that of the prediction set. This indicates a strong representativeness of the selected samples and contributes to the construction of a model with enhanced generalization capabilities.

### 3.3. UVE for Spectral Selection

**Figure 4:**
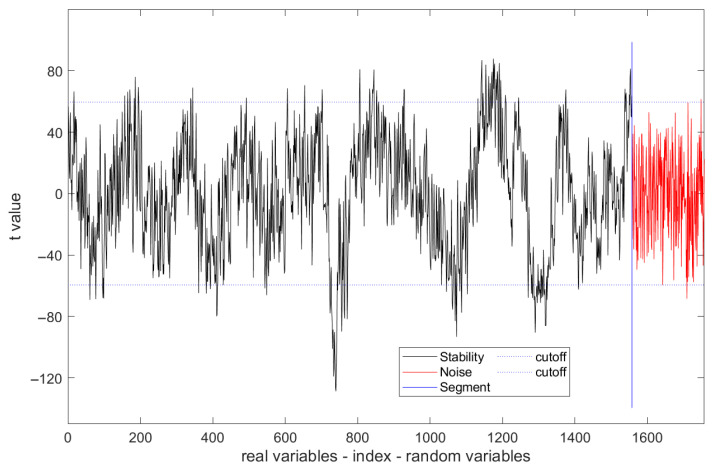
Stability of spectral variables validated by UVE selection methods.

Figure 4 shows the stability of spectral variables compared to the randomly added noise. Parameters of 200 noise variables, five k-folds, and a 99% confidence level were set in the cross-validation stage to optimize the combination of spectral variables. It can be seen that most variables fall between two stability limitation lines, and their distribution seems to lack regularity. Only those spectra whose absolute values were larger than the noise were regarded as the informative spectra (or called the characteristic wavelengths) and retained.

After extracting 86 characteristic wavelength variables using the UVE method, a PLS model was established to correlate the characteristic wavelengths with the TSS attribute. This model was compared with models that were established using the z-score method and raw spectral data. The results are shown in Table 2. The model based on raw spectral data exhibited the lowest prediction performance. Following preprocessing and feature wavelength selection, the Rp of the prediction set increased, while the RMSEP decreased, indicating that the model’s predictive accuracy for the prediction set was improved to a certain extent. Notably, the model obtained after UVE wavelength selection achieved the best performance among the three approaches. However, the overall improvement in model prediction performance after preprocessing and feature wavelength selection did not meet expectations, suggesting that further optimization is required for better results.

### 3.4. High-Frequency Variables Selected by the Successive Execution of UVE

**Figure 5:**
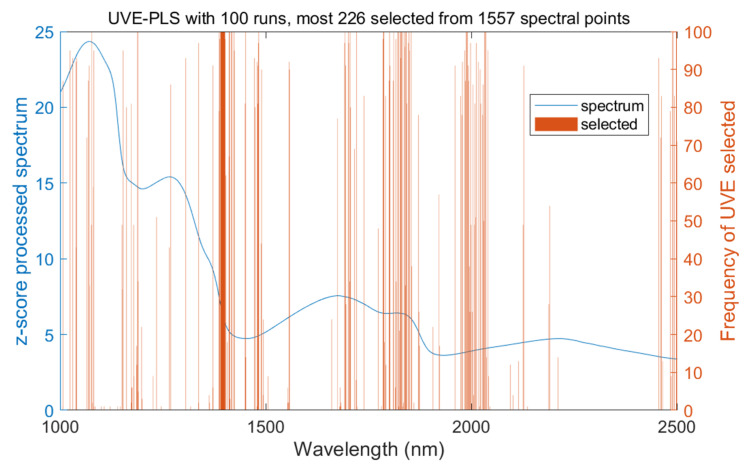
Distribution of spectral variables selected by UVE in 100 executions.

To maximize the selection of potential characteristic wavelengths associated with TSSs, the UVE method was used and repeatedly executed 100 times; finally, 226 wavelengths were obtained, representing approximately 19.5% of the total 1577 wavelengths analyzed. The selected spectral variables and their frequencies are visualized in Figure 5. The results reveal that the majority of these selected wavelengths are densely distributed near 1450 nm, 1800 nm, and 2000 nm, while other wavelength ranges are more scattered, which reflect the absorption characteristics of organic compounds in the fruit [20].

**Figure 6:**
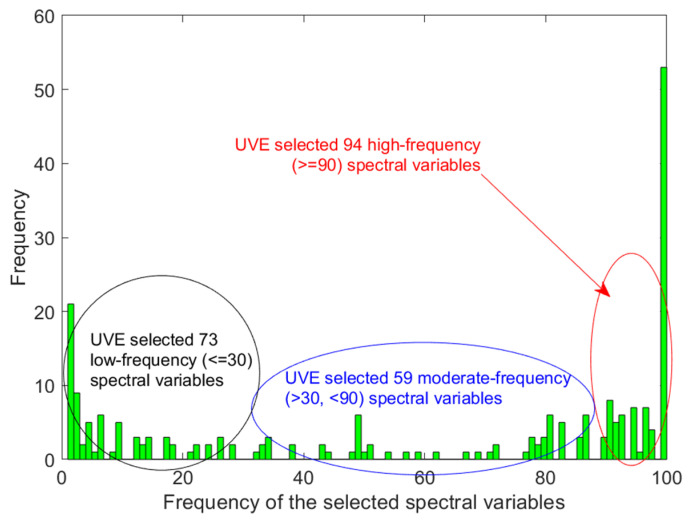
Distribution of high-frequency variables divided by their frequency.

The selected characteristic wavelengths from 100 runs of the UVE method showed a concentrated distribution in specific regions (as shown in Figure 5), indicating that certain wavelengths were frequently selected due to their strong association with TSSs. To qualify this relationship, a statistical analysis of the frequency of occurrence of all selected variables was conducted. Different characteristic wavelengths may contain different information, resulting in significant differences in the frequency of occurrence of different wavelengths. Based on their occurrence frequencies, wavelengths were categorized into three levels: ≥90, 30~90, and ≤30, as shown in Figure 6. Notably, nearly half of the spectral variables were selected more than 90 times, indicating that the UVE had consistent spectral selection regarding the fruits’ internal quality. This consistency suggests that the most frequently selected wavelengths can reliably reflect key features associated with TSSs, such as the absorption characteristics of C-H and O-H bonds in organic compounds, which is consistent with findings from related studies [18].

### 3.5. AdaBoost Ensemble from the Member Models

Based on the classified spectral variables, three PLS models (labelled as M1, M2, and M3) were established. Each model utilized a different subset of characteristic wavelengths corresponding to specific frequency tiers (≥90, 30–90, and ≤30 occurrences). The AdaBoost ensemble learning algorithm was then applied to dynamically adjust the weights of these member models. Statistical results are shown in Table 3 for the member models and the ensemble model. Among the three member PLS models, M1 and M2 exhibited similar performances during the calibration stage but turned to differences in predictive capacities. This difference suggests the robust predictive potential of models established with high-frequency selected wavelengths. The M3 model, constructed using wavelengths with frequency of less than 30, demonstrated the poorest performance in cross-validation among these three base models. However, it outperformed M2 in prediction accuracy on the prediction set., suggesting a complexity of interplay between model complexity and sample composition. Therefore, an ensemble model was proposed to integrate member (or base) models into a fusion (or ensemble) model that can reduce the impacts of adverse factors. The prediction deviation of these member models for apricot TSSs was calculated, and the initial error for weighting distribution were set as 1 and 1.5, respectively. Using the AdaBoost ensemble method, the weights for member models were close to 1/3 each, and the ensemble models demonstrated appropriate performance on the prediction set, indicating the stability of AdaBoost ensemble. Notably, compared to the model built using all selected wavelengths, the member model achieved a higher Rp and lower RMSEP for the prediction set, indicating enhanced predictive reliability. Further analysis revealed that the member models based on the subset with a frequency of occurrence ≥90% (M1) produced the best performance, with an RMSEP of 1.267 and an Rp of 0.883. This result suggests that these high-frequency wavelengths are strongly associated with TSSs.

**Figure 7:**
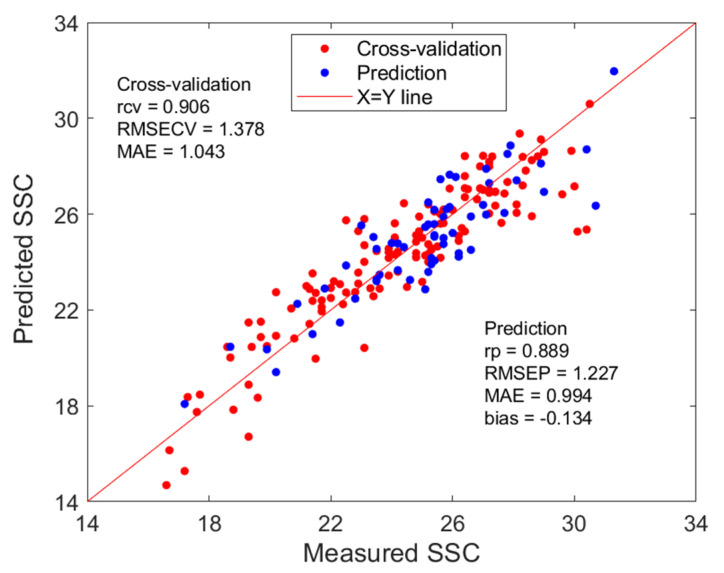
Scatter plot of measurements versus predictions by AdaBoost ensemble.

As can be seen from Table 3, after the AdaBoost ensemble learning and weighting allocation, the predictive performance of the ensemble model was further improved, with the model’s Rp reaching 0.889 and both RMSEP and MAE decreasing, as its scatter plot shows in Figure 7. Compared with the full-spectrum model and the UVE-selected variable model, the fusion model based on the characteristic wavelength subset (M1, M2, and M3) has certain advantages in predictive performance.

## 4. Conclusions

This study explored an AdaBoost ensemble model for predicting the internal quality of Xinjiang apricot by NIRS and the successive execution of UVE. After z-score preprocessing and removing the spectral outliers, the UVE method was successively executed to collect the potential wavelengths that correlated with TSSs. Through a frequency-based screen of the characteristic wavelength variables from 100 runs of UVE, it was found that using subsets of these characteristic wavelengths could help enhance the model’s predictive performance. Furthermore, the AdaBoost algorithm was employed to dynamically allocate weights to the member models of characteristic wavelengths, resulting in a further performance enhancement. The final ensemble model demonstrated superior predictive accuracy (Pearson’s correlation coefficient, Rp = 0.889) and generalization capability compared to single-model approaches, as validated by the reduced RMSEP and MAE. The findings of this research provide a practical method for the rapid and non-destructive assessment of apricot TSSs, which can be extended to the quality inspection of other fruits. Future research directions include exploring advanced ensemble learning methods to further optimize the prediction accuracy and robustness.

## Figures and Tables

**Table 1:** Stats of sample divisions for apricot TSSs.

	Number	Range (°Brix)	Mean	Std	CV (%)
Calibration	130	14.6~28.5	22.32	3.276	13.47
Prediction	59	15.2~29.3	23.10	2.704	10.77

Std: standard deviation of the samples’ attribute; CV: coefficient of variation.

**Table 2:** Comparison of different pretreatments by the PLS model.

Methods	Variables	LV	Calibration Set			Prediction Set			
			RMSECV	Rcv	MAE	RMSEP	Rp	MAE	Bias
Raw spectra	1557	12	1.452	0.886	1.107	1.338	0.870	1.082	−0.145
z-score pretreatment	1557	11	1.416	0.902	1.026	1.321	0.872	1.076	−0.047
Selected by UVE	86	12	1.381	0.907	1.065	1.323	0.872	1.035	−0.149

LV: latent variables in the PLSR model; PLSR: partial least squares regression.

**Table 3:** Results of RLS models based on high-frequency variables divided by their frequency.

Methods	Variables	LV	Calibration Set			Prediction Set			
			RMSECV	Rcv	MAE	RMSEP	Rp	MAE	Bias
Selected by UVE	86	12	1.381	0.907	1.065	1.323	0.872	1.035	−0.149
M1: Freq ≥90	94	10	1.374	0.907	1.029	1.267	0.883	1.014	−0.163
M2: Freq 30~90	59	12	1.373	0.908	1.058	1.309	0.873	1.071	−0.150
M3: Freq ≤30	73	10	1.392	0.899	1.086	1.269	0.881	1.046	−0.086
AdaBoost ^a^	/	/	1.378	0.906	1.043	1.267	0.889	0.994	−0.134

^a^ AdaBoost member weights: 0.3361, 0.3396, 0.3243.

## Data Availability

The data presented in this study are available upon request.
